# Optogenetic Induction of Pyroptosis, Necroptosis, and Apoptosis in Mammalian Cell Lines

**DOI:** 10.21769/BioProtoc.4762

**Published:** 2023-07-20

**Authors:** Kateryna Shkarina, Petr Broz

**Affiliations:** Department of Immunobiology, University of Lausanne, Lausanne, Switzerland

**Keywords:** Programmed cell death, Apoptosis, Pyroptosis, Necroptosis, Optogenetics

## Abstract

Regulated cell death plays a key role in immunity, development, and homeostasis, but is also associated with a number of pathologies such as autoinflammatory and neurodegenerative diseases and cancer. However, despite the extensive mechanistic research of different cell death modalities, the direct comparison of different forms of cell death and their consequences on the cellular and tissue level remain poorly characterized. Comparative studies are hindered by the mechanistic and kinetic differences between cell death modalities, as well as the inability to selectively induce different cell death programs in an individual cell within cell populations or tissues. In this method, we present a protocol for rapid and specific optogenetic activation of three major types of programmed cell death: apoptosis, necroptosis, and pyroptosis, using light-induced forced oligomerization of their major effector proteins (caspases or kinases).

## Background

Regulated cell death is a common feature of multicellular organisms and plays a key role in development and tissue homeostasis and in protecting the host against malignant growth and various pathogens. Research over the last two decades has identified over 12 different forms of regulated cell death (Galluzzi et al., 2018); however, their study is often complicated by the complex crosstalk and interconnectivity between the different cell death pathways (Bedoui et al., 2020). Additionally, the consequences of different types of cell death in the tissue still remain insufficiently understood, which is at least partially due to the challenges of selectively targeting single cells in multicellular populations, as well as to the pleiotropic effects of commonly used *natural* cell death triggers both on the dying cells and their neighbors.

**Figure 1.**
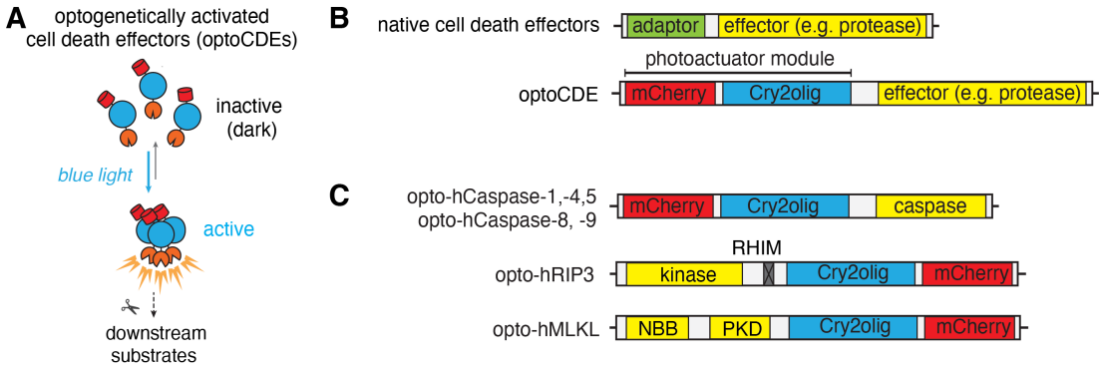
Schematic representation of the optogenetically activated cell death effectors (optoCDE) system. (A) OptoCDEs are inactive and monomeric in the dark state but are activated within seconds upon blue light illumination, which induces oligomerization of the Cry2olig photoactuator domain, activation of downstream substrates, and induction of cell death. (B) General architecture of major cell death effectors and design of optoCDE constructs. Cell death effectors used in the study generally consist of an adaptor domain (CARD or DED for caspases and RHIM for RIPK3) and an effector domain bearing protease (caspases), kinase (RIPK3), or membrane-disrupting (MLKL) function. In the optoCDEs, the effector domain is retained, while the adaptor is replaced with the Cry2olig-mCherry photoactuator module (mCherry is used to visualize optoCDE expression and/or clustering). **(C)** Schematic representation of major types of optoCDE constructs used in the study. The detailed description and evaluation of the optoCDE tools is available in the original paper (Shkarina et al., 2022). Figure adapted from Shkarina et al. (2022).

In recent years, multiple strategies have been developed to specifically ablate cells both in vitro and in living animals. However, some of these methods (such as laser ablation or photosensitization) still lack specificity regarding the type of cell death to be induced (Tirlapur et al., 2001; Qi et al., 2012), while others [such as chemically inducible dimerization of apoptotic or necroptotic effector proteins (Oberst et al., 2010; Wu et al., 2014)] suffer from a limited spatiotemporal control and require a delivery of soluble ligands, thus limiting their in vivo applications. To overcome these limitations and expand the scope of the tools available for programmed cell death induction, we recently developed a set of optogenetically activated cell death effectors (optoCDEs) (Shkarina et al., 2022), which enable selective induction of three major types of programmed cell death: apoptosis, pyroptosis, and necroptosis. These tools consist of three modules: 1) a photoactuator domain Cry2olig (Cry2 E490G), which responds to blue light by rapid homo-oligomerization, 2) an mCherry tag, which enables the detection of the cells expressing optoCDEs and estimation of the relative construct expression levels, and 3) an effector module (Figure 1A–1C). For opto-caspases, the effector module corresponds to the protease domain (p20 and p10 subunits) of corresponding caspases; the endogenous linkers and cleavage sites essential for the caspase activation are retained, while CARD (in caspase-1, -4, -5, -9, and -11) and DED (caspase-8) domains, responsible for the endogenous upper-level protein–protein interactions and homo-oligomerization, are removed. In opto-RIPK3, the design is similar, while the RHIM motif (responsible for the upstream interaction with the RIPK1) is mutated. In optoMLKL, the effector domain orientation in relation to Cry2olig and mCherry is reversed to keep the MLKL N-terminus (responsible for membrane binding and disruption) exposed. The considerations behind the construct design and testing of the different construct versions are described in more detail in the original paper (Shkarina et al., 2022). The blue light stimulation triggers the rapid activation and oligomerization of Cry2olig, which in turn results in the proximity-induced activation of effector domains and subsequent processing of downstream substrates, culminating in cell death. While Cry2olig alone responds to the blue light within seconds (Taslimi et al., 2014), the timing of cell death induction is defined by the kinetics of effector activation as well as availability and efficiency of the processing and activation of downstream substrates (such as apoptotic executioner caspases, necroptotic effector GSDMD, or pyroptotic effector MLKL); the first morphological features of cell death can usually be detected within minutes after the beginning of illumination.

The optoCDEs can be applied in vitro, as described in this protocol, as well as in vivo to selectively kill specific cells or cell populations in a highly controlled and specific manner (Shkarina et al., 2022). Additionally, the precise control over the illumination parameters, such as light intensity and duration, provides new means for cellular and mechanistic studies of these forms of cell death, as well as probing cell survival mechanisms that limit cellular damage downstream of these effectors.

## Materials and reagents

- **Cell culture and lentiviral transduction**
  1. Sterile serological pipettes (Falcon, catalog number: 357543)
  2. Sterile micropipette tips (Starlab TipOne, catalog number: S1120-8810)
  3. Tissue culture–treated cell culture flasks (TPP, catalog number: 90076)
  4. 10 cm Petri dishes (Falcon, catalog number: 351029)
  5. Syringes (5 and 10 mL) (Braun Omnifix, catalog numbers: 4616103V and 4616057V)
  6. Sterile tissue culture–treated 6-well flat-bottom plates (Eppendorf, catalog number: 0030720113)
  7. Sterile 50 and 15 mL Falcon tubes (SPL life sciences, catalog numbers: 50015 and 50050)
  8. Sterile 1.5 mL microcentrifuge tubes (Eppendorf, catalog number: 11.3817.01)
  9. Aluminum foil (Sigma, catalog number: 326852)
  10. 0.45 μm filters (Sarstedt, catalog number: 83.1826)
  11. Polybrene (Merck, catalog number: TR-1003-G)
  12. JetPRIME transfection reagent (Polyplus, catalog number: 101000027)
  13. HEPES 1M (Sigma, catalog number: H3375)
  14. Dulbecco’s phosphate-buffered saline (DPBS) 1× (Thermo Fisher Scientific, catalog number: 10010023)
  15. Dulbecco’s modified Eagle medium (DMEM) with GlutaMAX supplement (Thermo Fisher Scientific, catalog number: 10564011)
  16. RPMI 1640 medium, with GlutaMAX supplement (Thermo Fisher Scientific, catalog number: 61870044)
  17. Doxycycline hydrochloride (Sigma-Aldrich, catalog number: D3447)
  18. Puromycin (Invivogen, catalog number: ant-pr-1)
  19. Hygromycin B Gold (Invivogen, catalog number: ant-hg-1)
  20. LPS-B5 ultrapure (Invivogen, catalog number: tlrl-b5lps)
  21. PMA (Sigma-Aldrich, catalog number: P1585)
  22. Fetal bovine serum (Bioconcept, catalog number: 2-01F10-I)
- **Live-cell imaging and cell death detection**
  1. 8-well tissue culture–treated μ slides (ibidi, catalog number: 80826)
  2. Collagen solution from bovine skin (Sigma-Aldrich, catalog number: C4243)
  3. Opti-MEM reduced serum medium (Gibco, catalog number: 11058021)
  4. CellTox Green (Promega, catalog number: G8741)
  5. DRAQ7 (BioLegend, catalog number: 424001)
  6. Annexin V Pacific Blue (BioLegend, catalog number: 640918)
  7. Annexin V FITC (BioLegend, catalog number: 640906)
  8. Annexin V Alexa Fluor 647 (BioLegend, catalog number: 640912)
  9. CellEvent caspase-3/7 green (Thermo Fisher Scientific, catalog number: R37111)
- **Cell lysis and cytokine secretion analysis**
  1. Vision Plate 24, 150 micron, TC-treated, sterile (Life Systems Design, catalog number: 4ti-0241)
  2. 96-well flat-bottom plates
  3. Lactate dehydrogenase (LDH) cytotoxicity detection kit (Sigma, catalog number: 11644793001)
  4. Triton X-100 (Sigma, X100-500ML)
  5. Human IL-1β ELISA kit (R&D, catalog number: DY401)
  6. ELISA plates (Sigma-Aldrich, catalog number: M9410-1CS)
  7. LDH stop solution [2 M acetic acid (Sigma, catalog number: 64-19-7)] in dH_2_O, store at 4 °C
  8. Reagents and equipment for ELISA
  9. Reagents and equipment for the Western Blot analysis
  10. Fluorescence assay (Cisbio, catalog number: 62HIL1BPET)

## Equipment

- **For cell culture**
  1. Tissue culture hood (such as HERASAFE^TM^ KS, Thermo Scientific)
  2. Cell incubator (Forma^TM^ Steri-Cycle^TM^ CO_2_ Incubator, Thermo Scientific)
  3. Centrifuge (Eppendorf 5810R)
  4. Light microscope (such as Leica DMI6000B)
  5. Pipettes
- **For imaging**
  1. Point-scanning confocal (such as Zeiss LSM800 or Leica SP8)
- **For cell population–level assays**
  1. Light plate apparatus (Gerhardt et al., 2016) equipped with double row of 450 nm light LEDs
  2. Spectrophotometer/ELISA plate reader
  3. Multichannel pipettes
  4. Western blot imager

## Software

1. ZEN (ZEISS, https://www.zeiss.com/microscopy/en/products/software/zeiss-zen.html)
2. Iris (Jeff Tabor laboratory, http://taborlab.github.io/Iris/)
3. Fiji (NIH, https://imagej.net/software/fiji) (Version 2.3.0)
4. Software for the plate reader
5. Microsoft Excel (Microsoft, version 16.69.1)
6. GraphPad Prism (version 9.3.1.)

## Procedure

- **Generation of stable cell lines expressing optogenetically activated cell death effectors (optoCDEs)**
  1. Lentiviral particle production
    - Prepare stocks of purified lentiviral plasmids encoding optoCDE constructs. We recommend using endotoxin-free midi- or maxi-prep kits for plasmid purification.
    - Twenty-four hours prior to transfection, plate HEK293T cells in a tissue culture–treated 6-well plate at 5 × 10^5^ cells/well in 2 mL of fresh cell culture medium.
    - For each well, prepare the transfection mix, as summarized in Table 1, in 1.5 mL tubes. Mix gently and incubate at room temperature for 15 min.
      **Table 1.** Transfection mix per well of a 6-well plate

Components	Amount
pLVX-optoCDE	1.9 μg
PsPAX2	1.9 μg
VSVg	0.2 μg
JetPrime transfection reagent	5 μL
JetPrime Buffer	200 μL
    - Add the transfection mix dropwise to the cells. Ensure that the whole surface of the well is evenly covered (this can be assessed by monitoring the transient change of the medium color). Incubate the cells at 37 °C and 5% CO_2_.
    - At 6–12 h post transfection, replace the transfection mix with 2 mL of fresh cell culture medium for virus collection.
*Note: Use the appropriate cell culture medium for the cell line to be transduced.*
    - Incubate for an additional 36–48 h at 37 °C and 5% CO_2_.
*Note: Over the course of virus production, the transfected cells might change the morphology, round up, and lift, as a consequence of viral particle release.*
    - After 36–48 h, collect the virus-containing supernatants from transfected HEK293T cells into 15 mL Falcon tubes. At this step, the medium from the wells transfected with the same construct can be combined and further processed together.
    - Using a 5 or 10 mL syringe, gently pass the supernatants through a 0.45 μm syringe filter to remove cell debris and collect the filtrate into the clean 15 mL Falcon tube. Supplement the virus-containing supernatants with 20 μM HEPES (final concentration) to facilitate viral particle stability.
    - At this stage, the virus-containing supernatants can be either used directly or stored at -80 °C for several months.
  2. Cell transduction
    - Seed the appropriate number of cells to be transduced.
      i. For adherent cells: we recommend seeding 0.8 × 10^6^–1 × 10^6^ of cells per well of a 6-well plate, 6–24 h before transduction. Cells should be properly attached and cover 60%–70% of growth surface. The optimal seeding density can be adjusted to accommodate for the cell size and differences in the growth kinetics between different cell lines. We recommend using 2–5 mL/well of virus-containing medium for 6-well plates, 2 mL/well for 12-well plates, and 1 mL/well for 24-well plates.
      ii. For non-adherent cell lines: cells can be collected, centrifuged, and resuspended in the virus-containing medium immediately prior to transduction. The second centrifugation step (spinfection) can be performed either in plates or in 50 mL Falcon tubes (in this case, the cells need to be resuspended and transferred to the plates or flasks following the spinfection).
      iii. Include an additional well of mock-treated (polybrene-only treated) cells to control for cell viability and transduction efficiency.
    - Replace the culture medium with the virus-containing medium supplemented with 5 μg/mL polybrene to facilitate viral particle adhesion.
    - To facilitate the infection, centrifuge the cells with the virus at 3,000× *g* for 1 h at 37 °C. For the plate centrifugation, seal the sides of the plates with tape to prevent the accidental spillover of the supernatants during plate transfer and centrifugation. Suspension cells can also be centrifuged in 50 mL Falcon tubes. After the completion of this step, move the cells to the cell culture incubator.
    - After 6–12 h, aspirate the virus medium, wash cells 1–2 times with the pre-warmed DPBS, and add the appropriate volume of fresh growth medium. If cell density is too high, cells can be transferred into the larger vessel at this step.
    - Let the cells recover for 24–48 h to increase the cell number and allow for the proper expression of the selection markers and/or antibiotic resistance genes.
    - To select successfully transduced cells, replace the medium with fresh medium containing the appropriate antibiotic concentration.
      i. Use the non-transduced wells as the positive control for selection efficiency.
      ii. The optimal antibiotic concentration for each cell line can vary and has to be determined using an antibiotic titration.
      iii. For most cell lines, we have successfully used the selection medium containing 1–5 μg/mL of puromycin or 50–100 μg/mL of hygromycin B Gold.
    - After 3–5 days, remove the antibiotic-containing medium and expand the surviving cells.
  3. Cell line validation
    - To confirm the transduction efficiency, induce optoCDE expression by incubating the cells with 1 μg/mL doxycycline for 24–48 h (at this stage, cells need to be protected from light to prevent the optoCDE activation and cell death). Include the non-treated control (without doxycycline) to control for the leaky expression.
    - The percentage of mCherry-positive cells can be quantified using fluorescent microscopy or FACS. The efficiency of cell death induction can then be tested as described in sections B and C.
*Note: OptoCDEs are fused with mCherry allowing easy assessment of construct expression.*
    - Following selection and validation, the stable lines can either be frozen away in liquid nitrogen for storage or directly used for experiments.
- **Optogenetic induction and visualization of pyroptosis, necroptosis, and apoptosis using confocal microscopy**
  1. Cell preparation for imaging
    - One day before the experiment, seed the cells into the 8-well tissue culture–treated μ-slides at the concentration of 0.5 × 10^5^–1 × 10^5^ cells/well.
      i. If using other type of cell culture plates, the cell concentration should be adjusted to achieve 30%–70% of the density at the day of experiment. We recommend using a lower cell density for experiments focused on the high-resolution visualization of the dynamic cellular events during different types of cell death in adherent cells.
      ii. For poorly adherent cell types (such as HEK293T, MCF7, or equivalent), the slides can be additionally pre-coated with 5% collagen.
      1) Prepare the appropriate volume of 5%–10% dilution of bovine skin collagen in sterile PBS. Mix thoroughly by inverting. Avoid vortexing, as this may lead to the collagen precipitation.
      2) Add 100–150 μL of collagen solution per well. The volume of liquid should be sufficient to fully cover the growth surface.
      3) Incubate the slides at room temperature for 5 min; then, aspirate the collagen and air dry the slides for 30–60 min in the hood. During aspiration, avoid touching the bottom of the slide (growth surface), as this might lead to the disruption of the collagen layer and reduce cell adhesion.
      4) The coated slides can be stored at room temperature for several weeks.
    - To induce the construct expression in the cells, supplement the cell culture medium with 1 μg/mL doxycycline and incubate for 16–24 h. The longer induction time can be used to achieve higher expression levels but can also result in increased cytotoxicity.
    - To protect the cells from light following expression induction, construct a dark chamber by fully covering a petri dish with foil (Figure 2). Alternatively, cells can be placed in a plastic box or an alternative type of light-impermeable container, which allows air circulation and humidification.
      **Figure 2.**
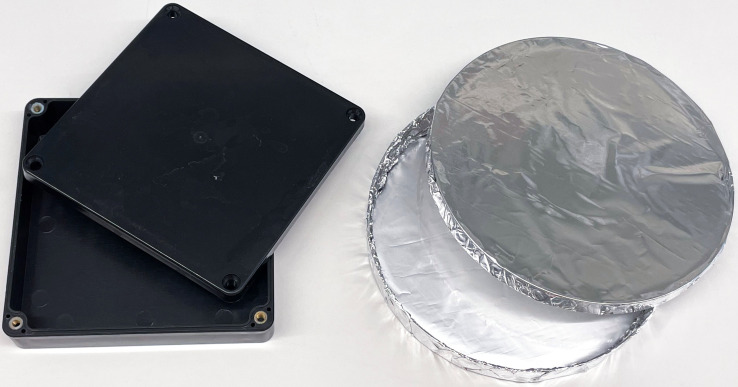
Examples of the chambers that can be used to protect optogenetically activated cell death effectors (optoCDE)-expressing cells from light
    - On the day of experiment: pre-heat the microscope to 37 °C. Ensure that the temperature is stabilized before the start of the imaging.
    - Gently wash the cells with pre-warmed PBS and replace the cell culture medium with pre-warmed Opti-MEM or alternative imaging medium. At this stage, all the manipulations need to be performed under dim light or red-light conditions to avoid spontaneous optoCDE construct activation.
    - Optional: for cell death detection, the following reagents can be directly added to imaging medium:
      i. Visualization of membrane permeabilization:
      1) CellTox Green: 1:10,000
      2) DRAQ7: 1:1,000
      ii. Visualization of PS exposure
      1) Annexin V Pacific Blue: 1:500
      2) Annexin V FITC 1:1,000
      3) Annexin V Alexa Fluor 647
      iii. Apoptotic caspase activation
      1) CellEvent caspase-3/7 green—it is recommended to pre-load the cells with the dye for 1 h before an experiment.
      2) Alternatively, one can utilize genetically encoded fluorescent protein-based caspase-3/7 reporters, such as VC3AI or ZipGFP.
  2. Microscope setup
    - Both 488 and 496 nm lasers can be used for Cry2olig activation. During continuous whole-field photoactivation experiments, Cry2olig stimulation can be coupled with simultaneous imaging of fluorophores in the green channel [in this case, it is preferable to set up this channel as the last in an acquisition sequence to obtain the *non-stimulated* (t = 0) images]. However, this is not possible when using pulsed activation and/or single-cell targeting; in this case, only red or far-red fluorescent dyes and proteins should be used for the visualization of cell death and other processes of interest.
    - Set up the time-lapse imaging. Keep in mind that Cry2olig activation will cumulatively depend on both the laser power and the frame rate, so both need to be adjusted accordingly (e.g., if using higher frame rate, it is recommended to decrease the 488 nm laser power, while longer frame intervals might require increased laser intensity for Cry2olig activation). As an example, Figure 3 shows the relationship between the blue laser intensity and the percentage of pyroptotic cells detected after 30 min of illumination.
      **Figure 3.**
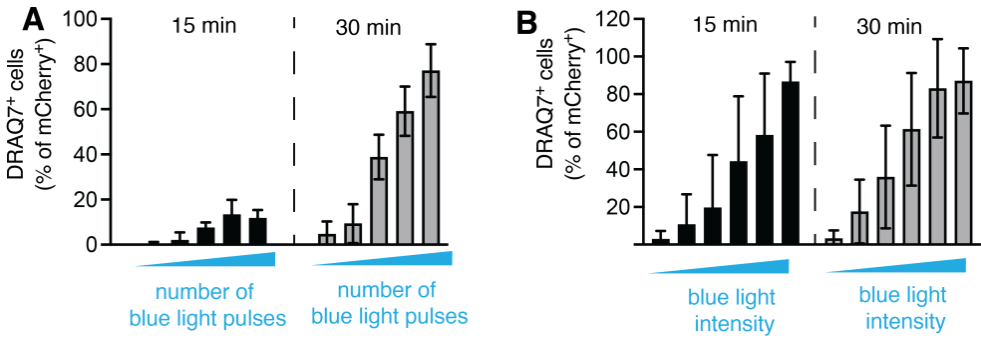
Relationship between opto-(h)caspase-1 activation and illumination parameters. (A) Quantification of pyroptotic (DRAQ7^+^) cells at 15 and 30 min post transient blue light illumination, and (B) at continuous repetitive (every 15 s) stimulation with blue light of various intensity. The data corresponds to Figure 3A–3B in the original paper (Shkarina et al., 2022).
    - Acquire the time-lapse series. An example of the typical pyroptotic, necroptotic, and apoptotic cell morphology is shown in Figure 4. Note that the kinetics and morphological characteristics of each type of cell death vary among cell types and might also depend on the optoCDE construct expression level.
      **Figure 4.**
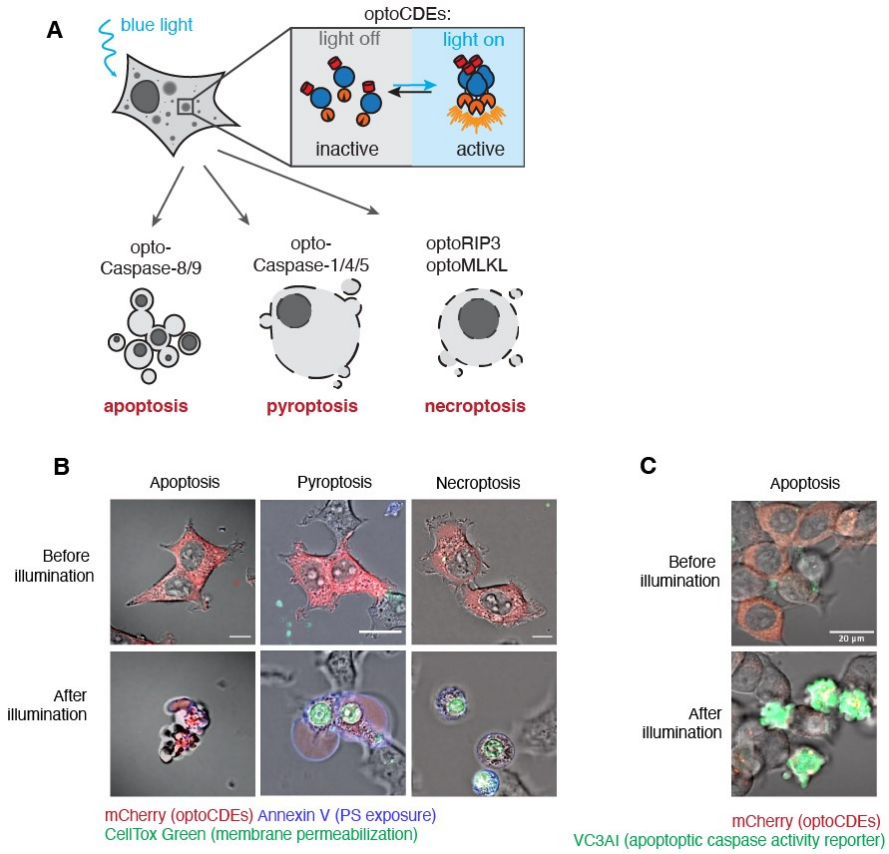
Assessment of optogenetically activated cell death effectors (optoCDE)-induced cell death using microscopy. (A) Schematic representation of expected morphological changes in cells undergoing different types of cell death (apoptosis, pyroptosis, and necroptosis) in response to light-induced optoCDE activation. (B) Representative morphological features of HEK293T cells expressing pyroptotic [opto-(h)caspase-1], apoptotic [opto-(h)caspase-8], or necroptotic [opto-(h)RIPK3] effectors and stimulated with blue light. Red: mCherry-tagged optoCDEs; green: CellTox Green; blue: Annexin V. Due to the differences in cell death kinetics, the *after illumination* time point refers to 30 min for pyroptotic, 60 min for apoptotic, and 90 min for necroptotic cells. The images are derived from the original paper (Shkarina et al., 2022). (C) Representative images of cells expressing genetically encoded caspase-3/7 reporter VC3AI (green) and undergoing light-induced apoptosis approximately 1 h after illumination.
    - Additionally, when doing this type of experiments for the first time, perform a similar experiment with the same cell type expressing Cry2olig alone and monitor the signs of cell death and/or abnormal changes in the cellular behavior related to the phototoxicity. If such changes are observed, decrease the laser power and/or the frame rate.
  3. Optogenetic induction of pyroptosis in single cells
    - Set up the region of stimulation (ROI) corresponding to the specific cytoplasmic region in the cell to be targeted using ZEN “Bleaching” and “Regions” mode.
      i. To account for the light diffusion and minimize the influence on the neighboring cells, the ROI should not exceed 5–10 μm^2^.
      ii. The optimal laser intensity and number of scanning iterations varies depending on construct expression level, cell type, and type of cell death, and has to be determined empirically.
      iii. If the signs of cell death (such as membrane blebbing, DRAQ7, or Annexin V positivity) are observed in the neighboring cells, reduce the ROI size and/or laser intensity or number of scanning iterations.
    - Set up the time-lapse experiment. This should include several frames before the photoactivation as the baseline, after which the ROI stimulation is performed, and additionally 30–60 min or more after.
    - Acquire the time lapse. To monitor for cell death, the imaging medium can be supplemented with the far-red cell death dyes (Annexin V or DRAQ7). The example of such experiment is shown at Figure 5.
      **Figure 5.**
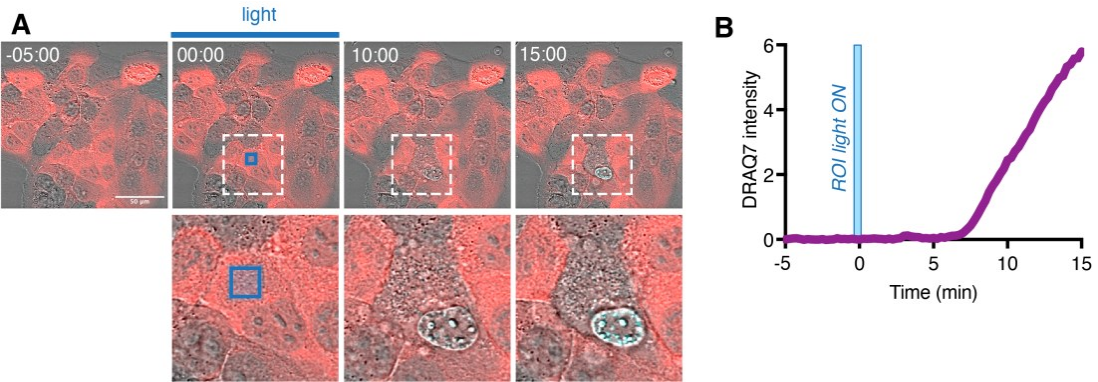
Single-cell optogenetic induction of pyroptosis. (A) Representative time-lapse images of HaCaT cells, where the cytoplasmic region of a selected single cell (inset) is selectively photoactivated with blue light [blue square represents region of illumination (ROI)] at 0 min. Note the morphological changes and gain of DRAQ7 signal (turquoise) in targeted cell but not in the neighboring cells. (B) Quantification of DRAQ7 intensity in the nucleus of the photoactivated cell. Figure adapted from Shkarina et al. (2022).
    - If signs of optoCDE activation and cell death are also detected in the neighboring cells, repeat the experiment with the reduced laser power and/or number of pulses or decrease the ROI area.
  4. Sub-lethal opto-caspase-1 activation
    - Set up the ROI stimulation experiment as described above. In this experiment, the ROI can include the whole field of view or be limited to the single cell.
    - Reduce the 488 nm laser power to 0.1%–0.2% and number of iterations to 1–3.
    - To monitor membrane permeabilization, supplement the imaging medium with DRAQ7. Use higher dye concentration (1:100–1:500) and laser power/gain at this stage to detect low amount of membrane damage.
    - Perform the imaging and photoactivation. The transient low-level optoCDE activation will likely lead to three phenotypes: 1) the cells that will undergo cell death (acquire “high” DRAQ7 staining); 2) the *survivor* cells (might acquire moderate DRAQ7 staining and transiently display some early features of cell death, such as membrane blebbing or nuclear condensation, but are able to revert to the normal morphology after), and 3) the cells that are not affected by the stimulation (not gaining DRAQ7 signal and no change in morphology) (Figure 6). The ratio of these cells in the population might depend on the optoCDE expression level, as well as additional cell-intrinsic factors (such as the variability in expression of the downstream effectors or activity of membrane repair systems).
    - Adjust the stimulation parameters to achieve the desired optoCDE activation and cell survival ratio and repeat the experiment.
    **Figure 6.**
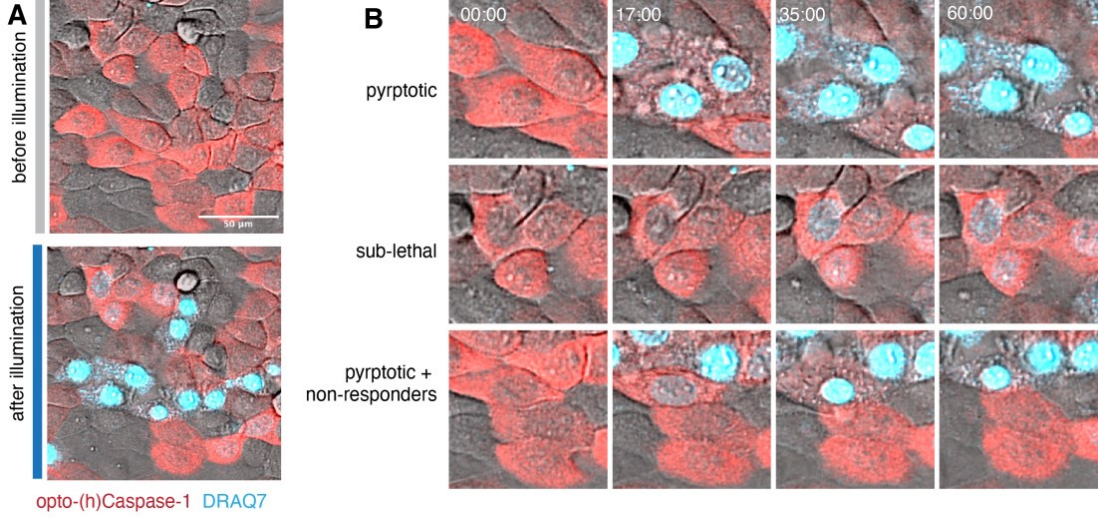
Sub-lethal induction of pyroptosis using transient opto-(h)caspase-1 activation in human keratinocytes. The confluent monolayer of HaCaT cells was transiently stimulated with low-intensity (0.2 mW/cm^2^/pulse, three pulses) blue light at 3 min after the beginning of time lapse, and the data was acquired for 60 min. (A) Representative images of the whole illuminated population before (at 0 min) and after (60 min). DRAQ7 (turquoise) is a membrane-impermeable DNA-binding dye used to visualize pyroptotic membrane permeabilization. (B) Close-up images of cells displaying three types of fates: pyroptotic (top), sub-lethal (middle), and both pyroptotic and non-responding cells (bottom). Note the strong DRAQ7 signal and loss of cytoplasmic mCherry in pyroptotic cells and low DRAQ7 positivity in the sub-lethally activated cells.
- **Optogenetic induction of pyroptosis in human macrophage-like cell lines**
  **Important points**
  1. In this protocol, we utilize U937, human monocyte-like cell line, which can be differentiated into the macrophage-like phenotype using PMA treatment. However, a similar type of experiments can be performed with other cell lines.
  2. Prior to seeding, transgenic U937 lines are grown in suspension in T75 flasks in complete RPMI medium. For optimal growth, the medium is exchanged every 2–3 days. Avoid growing the cells to too high density, as this will lead to the reduction of cell viability and might impact the differentiation.
  3. The light plate apparatus was manufactured, assembled, and calibrated as described previously (Gerhardt et al., 2016). The programming of the devices is performed using Iris (http://taborlab.github.io/Iris/).
  4. To determine the optimal illumination parameters for each type of cell death and for each cell type, we recommend testing a range of light intensities and illumination duration. When doing the experiment for the first time, always include the wild-type cells and/or cells expressing Cry2olig alone to control for the phototoxicity.
  5. This protocol describes the analysis of pyroptosis induction using opto-(h)caspase-1; however, a similar procedure can be used to activate other optoCDE constructs.
  **Step-by-step protocol**
  1. Cell seeding and differentiation
    - Collect the desired volume of U937 cells into the 50 mL flasks and centrifuge at 300× *g* at room temperature for 5 min. Discard the medium.
    - Resuspend the cells in fresh RPMI.
    - Count the cells and resuspend to the final density of 250,000 cells/mL.
    - Add PMA to the final concentration of 5 ng/mL.
    - Seed in 24-well black tissue culture–treated plates (4titude). Include additional wells for the non-illuminated cells and the positive (total lysis) control.
    - After 24 h, remove the PMA-containing medium, wash once with pre-warmed PBS, and add fresh growth medium.
    - After 48 h, replace the growth medium with the induction medium containing 2 μg/mL doxycycline and incubate cells overnight to induce optoCDE expression.
  2. Illumination
    - Set up the light plate apparatus in the cell incubator (Figure 6B). If using transient illumination times, these experiments can be performed on the bench before moving the plates to 37 °C.
    - Generate the custom illumination program using IRIS software. The example of an illumination layout is shown in Figure 7C. We recommend using duplicate wells for each illumination condition and including additional wells for the non-illuminated controls and total cell lysis (cells treated with 1% Triton X-100).
      **Figure 7.**
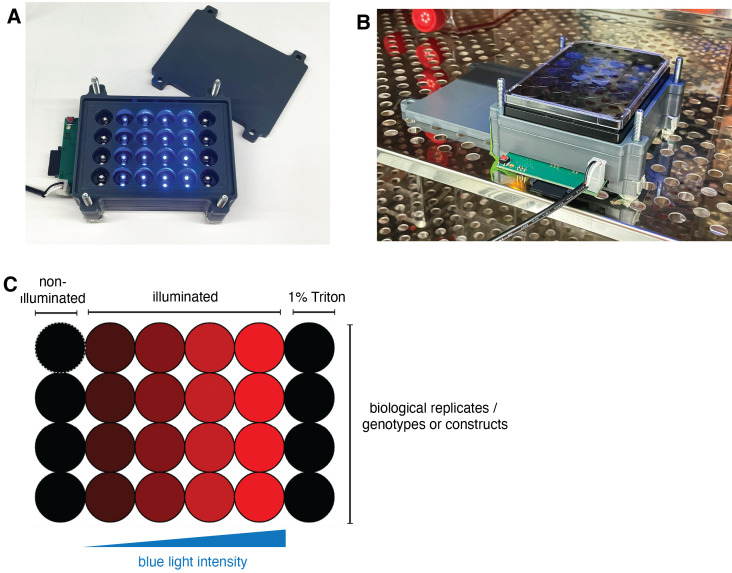
Light plate apparatus setup. (A) Photo of the self-made light plate apparatus device used for the lactate dehydrogenase (LDH)/ELISA and western blot assays. (B) Setting up the device in the cell culture incubator for long-term experiments. (C) Example of the illumination layout used for determining the optimal illumination parameters.
    - Download the program and transfer the files to the micro-SD card. Perform a short test illumination round to ensure that the device works properly.
    - Remove the cell culture medium from the cells and add 300 μL of pre-warmed Opti-MEM or equivalent serum-free phenol red free medium per well.
    - Place the plate into the light plate apparatus, cover the lid, and start the illumination. Upon the end of the program, the lights should turn off automatically.
    - Remove the plate from the light plate apparatus. Add 30 μL of 10% Triton X-100 to the positive control (total cell lysis) wells. Using a P1000 pipette, pipette 5–10 times to lyse the cells.
    - Centrifuge the plate at 500× *g* for 5 min at room temperature to pellet the cell debris.
    - Gently collect the supernatant from each well to the pre-labeled 1.5 mL microtubes. Avoid touching the well bottom to prevent debris collection. At this stage, 100 μL of supernatant can be used for lactate dehydrogenase (LDH) measurement, while the rest can be preserved at -20 °C for the cytokine measurement.
  3. Assessment of cell lysis using LDH activity assay
    - Prepare the appropriate volume of the LDH reaction mix.
    - Using a multichannel pipette, add 30 μL of reaction mix per well of a flat-bottom 96-well plate. Avoid creating bubbles, as this will impact the assay efficiency and reading.
    - In each well, add 30 μL of the cell supernatants collected in the step 2h. Include two to three technical replicates per each experimental well. Minimize delays while pipetting, as this might impact the difference in the colorimetric reaction between the wells and lead to bias.
    - Mix by gently tapping the plate on the side. Avoid mixing by pipetting, as this will generate bubbles. If bubbles are produced during pipetting, they can be manually removed before reading the plates using either syringe needles or an inverted Bunsen burner.
    - Incubate the plate for 15–20 min at room temperature, protected from light.
    - Add 30 μL of stop solution per well and gently mix.
    - Measure the absorbance using a 490–492 nm filter.
  4. Assessment of the IL-1β release
    - IL-1β release quantification following illumination is typically quantified using ELISA, performed according to the manufacturer’s protocol (available at R&D website).
    - We recommend using several dilutions (1:1, 1:2, 1:5) of the collected supernatants, as the amount of IL-1β in undiluted supernatants frequently exceeds the dynamic range of assay.
    - Alternatively, a FRET-based no-wash homogeneous time resolved fluorescence assay can also be utilized for quicker single-step IL-1β quantification.
  5. Western blot analysis of optoCDE activation and cell death
    - Before the start of the experiment, prepare the following reagents:
      i. Pre-labeled 1.5 mL microtubes for supernatant and lysate collection.
      ii. Cell lysis (RIPA or equivalent) and loading buffer.
      iii. Reagents for phenol-chloroform protein precipitation.
      iv. Reagents for the polyacrylamide gel preparation (or pre-cast gels).
      v. Western blot running, transfer, and wash buffers.
    - Perform the illumination of the plates as described in step 5a. Ensure that the cells are in the good condition before the start of experiment and are mCherry-positive (this can be assessed using epifluorescent microscopy).
    - Centrifuge the plates at 300–500× *g* for 5 min at room temperature to pellet the dead cells and collect the supernatants from each well into 1.5 mL microcentrifuge tubes.
    - Immediately add 50–70 μL of hot (95 °C) loading buffer to the remaining adherent/pelleted cells. At this stage, plates can be processed immediately or sealed with parafilm and stored at -80 °C for later processing.
    - Pipette the lysis buffer up and down 5–10 times and collect the lysates into the microtubes. If lysates become too viscous at this stage, they can be additionally sonicated.
    - Incubate for 5 min at 95 °C.
    - In parallel, perform the protein precipitation in the cell supernatants using the phenol-chloroform protein extraction method (Demarco et al. 2022). After the precipitation, the dried protein pellets can be either resuspended in 1× lysis/loading buffer and processed independently or combined with lysates of corresponding wells.
    - Load samples on the 10%–12% polyacrylamide gels.
    - Perform the gel running and western blot analysis of the samples following the protocol available in the host lab. The detailed protocol for western blot analysis of such samples can also be found in Demarco et al. (2022).
    - For the detection of inflammatory opto-caspase activation, we recommend using the following primary antibodies: rabbit anti-cleaved IL-1β (83186, CST; 1:1,000), which detects IL-1β by activated opto-(h)caspase-1; mouse anti-IL-1β (12242, CST; 1:1,000), which detects full-length unprocessed IL-1β in non-activated cells; rabbit anti-GSDMD (ab210070; 1:1,000; Abcam), which detects non-activated GSDMD in resting cells; rabbit anti-cleaved N-terminal GSDMD (ab215203; 1:1,000; Abcam), which detects active GSDMD cleaved by opto-(h)caspase-1; mouse anti-caspase-1 (clone Bally-1 AG-20B-0048-C100; 1:1,000; AdipoGen); mouse anti-mCherry (ab125096; 1:2,000; Abcam); and HRP-conjugated mouse anti-tubulin (ab40742; 1:5,000; Abcam), used as a loading control. The secondary HRP-conjugated or fluorescently labeled antibodies can be used according to the lab’s choice. A representative blot is shown in Figure 8.
  **Figure 8.**
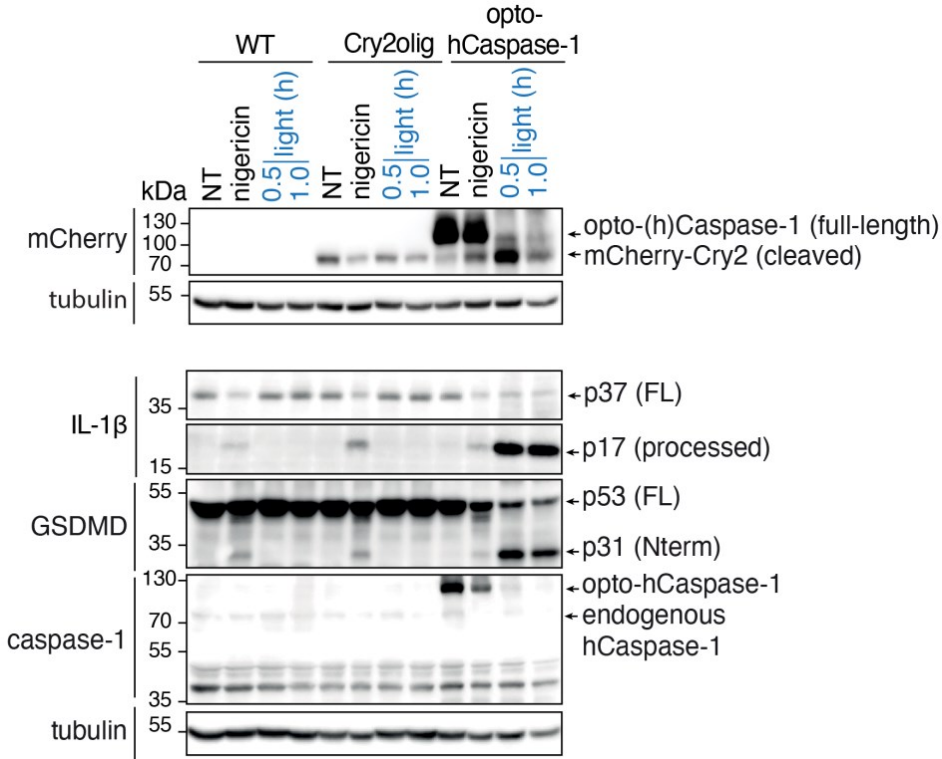
Example of the western blot analysis of opto-caspase-1-induced pyroptosis in U937 cells. Experiment was performed as described above and treatment with nigericin, an NLRP3 inflammasome activator, was used as a positive control to detect IL-1β and GSDMD processing. Note the cleavage of opto-caspase-1 (as detected by the disappearance of full-length opto-caspase-1 band and accumulation of Cry2olig-mCherry), IL-1β, and GSDMD upon illumination. Figure adapted from Shkarina et al. (2022).

## Data analysis

1. **Analysis of microscopy images**
  The analysis of the microscopy images is described in the Methods section of the original publication (Shkarina, et al., 2022). For quantification of different types of cell death, we assessed several parameters: a) appearance of the characteristic morphological changes associated with the each type of cell death (cell rounding and swelling, nuclear condensation for pyroptosis and necroptosis, nuclear fragmentation and persistent membrane blebbing for apoptosis); b) Annexin V staining, as an indicator of membrane scrambling and phosphatidylserine exposure (for all three types of cell death); and c) uptake of specific dyes associated with the loss of membrane integrity, such as CellTox Green or DRAQ7 (for pyroptosis and necroptosis). Additionally, activation of the apoptotic caspases can be monitored using genetically encoded or chemical fluorogenic caspase reporters (such as VC3AI, described in the original paper, or CellEvent caspase-3/7 reporter system). The number of dying cells can be normalized to the total number of cells per field of view, or, alternatively, to the number of mCherry-positive cells in the population, to account for the construct expression and transduction efficiency.
2. **Analysis of LDH assay and ELISA results**
  LDH release from pyroptotic (or generally necrotic) cells following illumination is quantified using the following equation:
  (LDHsample - LDHnegative control)/(LDHpositive control - LDHnegative control) × 100
  Where negative control is assay medium (e.g., Opti-MEM or other phenol red free medium) and positive control corresponds to the cells lysed with the 1% Triton X-100.
  Quantification of IL-1β release is performed according to the manufacturer’s protocol. Note that the variation in initial (pre-illumination) cell density between the cell lines or conditions can have a strong effect on the amount of detected IL-1β. This can be corrected by normalizing IL-1β values to the ratio of maximum LDH values from lysed cells. An example of the LDH and IL-1β secretion data obtained from this type of experiments is shown in Figure 9.
  **Figure 9.**
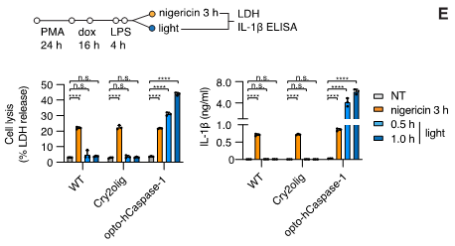
Example of lactate dehydrogenase (LDH) release and IL-1β secretion upon illumination-induced opto-(h)caspase-1 activation. Nigericin (NLRP3 inflammasome activator) was used to monitor cell competency to endogenous inflammasome activation. Figure adapted from Shkarina et al. (2022).

## Notes

**General notes**

1. When inducing different modes of cell death, it is important to take into consideration the selection of the appropriate cell line/type. We observed that the ability of cells to undergo different forms of cell death upon optoCDE activation varies among the different cell lines and cell types tested. This might depend on several factors:
  - Effector efficiency: we observed that, due to the differences in substrate processing kinetics and efficiency between different caspases, and also kinetic and mechanistic differences between separate cell death modalities, it might be necessary to adjust the levels of construct expression or stimulation to achieve similar cell death levels. As an example, in some cell lines, achieving an equal level of apoptosis might require higher expression of more prolonged stimulation for opto-caspase-8 than opto-caspase-9. Also, the same is applicable for opto-caspase-4 vs. opto-caspase-1 vs. opto-caspase-5, and for optoRIP3 vs. optoMLKL (although the last case can be explained by MLKL being the most distal cell death effector in necroptosis, while RIP3-induced necroptosis would be initiated more proximately and constrained by both endogenous MLKL levels and post-translational regulation).
  - Expression of downstream effectors, such as GSDMD for inflammatory (pyroptotic) caspases or MLKL for optoRIPK3. These proteins can be co-expressed with optoCDEs to achieve pyroptosis in the cell lines that are either naturally deficient (such as HEK cells) or express low levels (HeLa) of them endogenously.
  - Downstream regulatory mechanisms (such as phosphorylation or other types of post-translational modifications or protein–protein interactions), which might limit or promote optoCDE activation in some cell types.
2. Spontaneous or unwanted optoCDE activation due to overexpression or low-level activation due to visible light exposure might induce cytotoxicity or lead to the negative selection of expressing cells. Thus, it is essential to always protect the cells from visible light following the induction of optoCDE construct expression, or when using constitutive expression systems. All necessary cell handling, such as treatments or medium exchange, should be performed under dim light conditions, or using lab space and hoods equipped with red-light sources (lamps or LED strips). Additionally, we strongly recommend using inducible expression systems (such as Tet-ON) and tetracycline-free serum for stable cell line generation and maintenance.
3. Also, avoid checking the expressing cell lines under the microscope using violet, blue, or transmitted (white) light before the beginning of the experiment, as this will trigger the optoCDE construct activation. Usually, the mCherry expression in stable lines can be used as a good proxy measurement of the viability in unstimulated cells. Alternatively, visual assessment of cell density and morphology can be performed in separate wells, which can then be excluded from the experiment.
4. We recommend determining the optimal illumination parameters for each optoCDE construct and each cell line by testing several different blue light intensities and/or illumination duration. Additionally, it is essential to include wild-type cells and cells expressing Cry2olig alone to control for phototoxicity and potential non-specific effects of Cry2olig overexpression and photoactivation.
